# The association between physician salary and competitiveness of that specialty in the match: money still matters

**DOI:** 10.1093/fampra/cmaf021

**Published:** 2025-05-12

**Authors:** Mark H Ebell, Julie P Phillips

**Affiliations:** Department of Family Medicine, College of Human Medicine, Michigan State University, 804 Service Rd, East Lansing, MI 48824, United States; Department of Family Medicine, College of Human Medicine, Michigan State University, 804 Service Rd, East Lansing, MI 48824, United States

**Keywords:** specialty, primary care, physician salary, residency training

## Abstract

**Introduction:**

Given high levels of student debt and a desire for high income, we hypothesize that the mean salary of a medical specialty is correlated with how desirable that specialty is for graduating US medical students.

**Methods:**

We used salary data from a 2024 survey of 33,000 US physicians. As a proxy for desirability or competitiveness, we used the percentage of year 1 positions filled with US allopathic seniors based on data from the National Residency Match Program. Scatter plots were created and Pearson correlation coefficients were calculated.

**Results:**

There was a strong positive correlation between salary and competitiveness for US allopathic seniors (*r* = + 0.65). A negative correlation was seen for US osteopathic seniors (*r* = −0.53) and international medical graduates (*r* = −0.58).

**Conclusions:**

A specialty’s salary is strongly associated with its competitiveness for US allopathic seniors. Data for osteopathic seniors and international graduates shows the opposite association, suggesting a channeling bias of these students into lower-paying specialties or more successful efforts to encourage primary care careers.

**Key messages**

- There continues to be a strong positive correlation between a specialty’s salary and its competitiveness among US allopathic graduates.
- On the other hand, a negative correlation was seen for US osteopathic graduates and international medical graduates.
- These findings require further study to understand them, although a potential explanation is bias against osteopathic and IMGs among high-salary specialty training programs.

## Introduction

Compared with other countries, the United States has an underdeveloped primary care work force. A report from the Commonwealth Fund found that US adults were the least likely of 10 highly resourced economies to report having a longstanding relationship with a primary care physician [1]. This is concerning because the supply of primary care physicians in the United States is measurably correlated with population mortality [2].

US medical students have high levels of debt by the time they graduate, approximately $200,000 in 2023. These high levels of debt are an important potential motivator to have a higher salary [3]. Thus, we hypothesize that high-paying specialties will be more desirable to medical students than lower-paying primary care specialties, and thus more competitive in the national residency match process.

This is the fourth in a series of analyses dating back to 1989 examining the association between physician salary for a specialty and the competitiveness of that specialty in the National Residency Match Program (NRMP), using the percentage of US allopathic seniors matching to each specialty as a proxy for competitiveness [4–6]. We will also compare that association with the associations for US osteopathic seniors and international medical graduates.

## Methods

Physician salary by specialty was based on self-reported salaries from 33,000 US physicians in a survey by the medical social network Doximity [7]. Previous articles in this series used data from the Medical Group Management Association, but this is no longer available without a subscription. Doximity was chosen because it has over 80% of US physicians as verified members, making it likely to be representative [8]. As a proxy for “competitiveness” we used the percentage of PGY-1 residency positions filled by US allopathic seniors, using data from the NRMP for 2024 [9]. The proportions of US osteopathic seniors and international medical school seniors were also analyzed for comparison. All analyses were performed using Stata version 18.5 (StataCorp, College Station, Texas). Scatter plots were created using the “twoway scatter” command the “correlate” command was used to estimate Pearson’s correlation coefficient (*r*).

## Results

**Figure 1.**
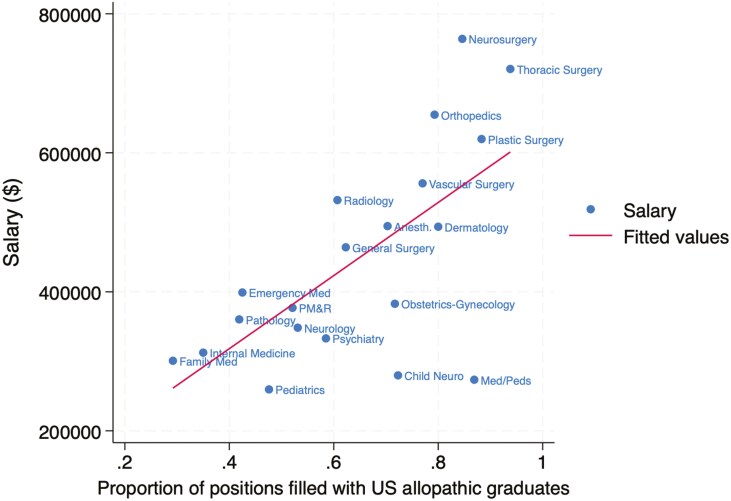
Association between mean physician salary and percentage of first-year residency positions filled by US allopathic seniors in the Match.

**Figure 2.**
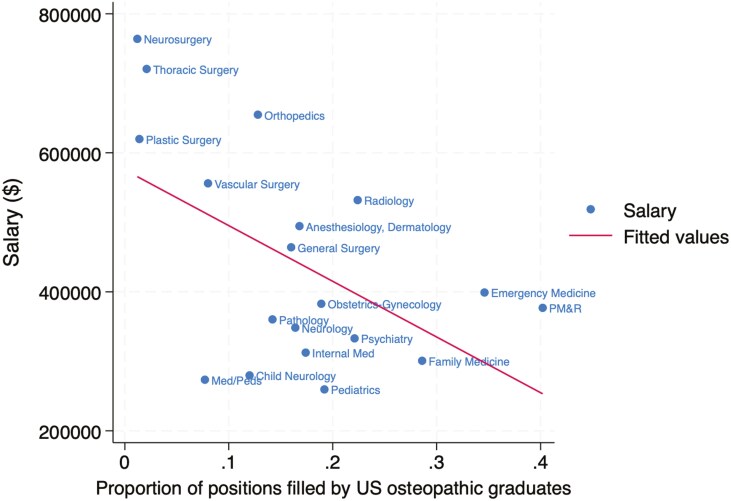
Association between mean physician salary and percentage of first-year residency positions filled by US osteopathic seniors in the Match.

**Figure 3.**
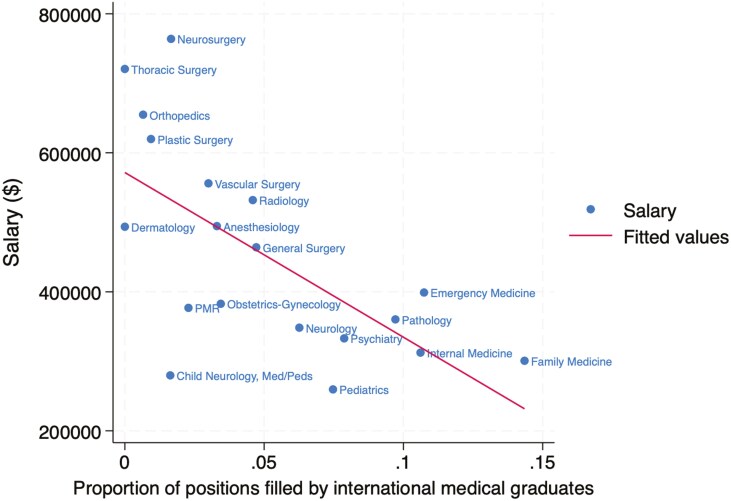
Association between mean physician salary and percentage of first-year residency positions filled by international medical graduates in the Match.

**Figure 4.**
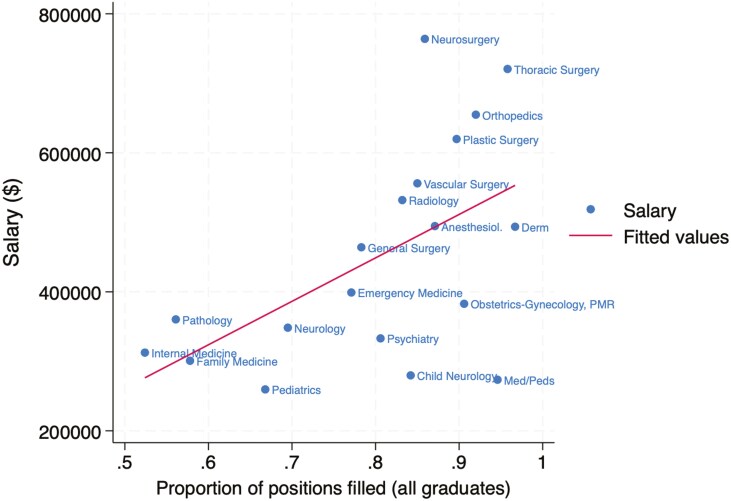
Association between mean physician salary and the percentage of first-year residency positions filled by all medical graduates in the Match.

The association between specialty salary and competitiveness in the match is summarized for US allopathic seniors in Fig. 1, US osteopathic seniors in Fig. 2, and international medical graduates in Fig. 3. The association for all groups combined is shown in Fig. 4. The Pearson correlation coefficients were + 0.65 for US allopathic graduates, −0.53 for US osteopathic graduates, −0.58 for IMGs, and + 0.51 for all groups combined. This compares with correlation coefficients of + 0.85 in 1989 [4], 0.82 in 2008 [5], and + 0.71 in 2016 [6] for US allopathic graduates.

## Conclusions

There continues to be a strong positive correlation between the average salary in a specialty and the competitiveness of that specialty as measured by the percentage of positions filled by US allopathic medical school seniors. This association is similar to that seen in previous reports. There has been a gradual decline in correlation coefficients from 1989 to the present report. This suggests that the association between salary and specialty, while still strong, may be weakening. The reason for this is not clear but may represent a greater value on non-financial rewards of a specialty and a desire for greater life balance.

However, inverse correlations were seen for US osteopathic seniors and IMGs. One possible explanation for these inverse correlations is a channeling of those graduates into lower-paid primary care specialties and conversely, lower match rates in highly compensated subspecialties. It suggests a possible bias against osteopathic graduates and IMGs that is not warranted by the data on their performance. In fact, an observational study found significantly lower mortality among older hospitalized Medicare patients cared for by IMGs (11.2% vs 11.6%) and no differences in readmission rates [10]. Other studies have found no difference between allopathic and osteopathic physicians with regards to outcomes in hospitalized patients [11], outpatient visit duration, and delivery of preventive services [12].

Another possible explanation for the inverse correlation between salary and competitiveness for osteopathic graduates is that osteopathic medical schools do a better job of selecting primary care-oriented students and emphasizing primary care in their curricula, resulting in more graduates selecting primary care specialties (despite their lower salaries). This suggests that allopathic educators could learn how to foster an interest in primary care from their osteopathic colleagues.

A high-functioning health system needs a sufficient supply of excellent primary care physicians. Factors such as high levels of medical student debt, a high degree of administrative burden, lower salaries than many other specialties, and long hours drive medical graduates away from primary care specialties. There are currently 67 primary care physicians per 100,000 Americans, compared with 133 per 100,000 Canadians. Only 34% of US physicians are in outpatient primary care, and this pipeline has declined significantly, with only 15% of physicians entering outpatient primary care practice in 2021 [13]. An important driver of students away from primary care is likely the large wage gap between primary care and subspecialty physicians in the United States, which is much higher than in other countries. For example, in the UK a general practitioner makes approximately 82% as much as a specialist, compared with the much larger gap shown in Figs 1–4 [14]. For example, radiologists and dermatologists earn approximately twice as much as family physicians in the USA.

The only way to reduce this wage gap is to increase primary care physician salaries (which are already higher than in other wealthy countries) or to reduce the salaries of subspecialists. The USA is also different from many other countries in that the number of residency positions for a specialty is not allocated centrally but rather is largely driven by the needs of regional or local health systems and hospitals. Effective primary care that keeps patients out of hospitals may not be aligned with the financial incentives of these health systems.

Another factor central to reimbursement is the Resource Based Relative Value Scale, which determines how much each physician activity is reimbursed. It is determined by the Specialty Society Relative Value Scale Update Committee which has a representative from each specialty, meaning that procedural specialties and subspecialties vastly outnumber primary care specialties on this panel [15]. Reform of the panel to shift power away from specialist physicians and to other stakeholders including more primary care physicians, payors, patients, and other members of the healthcare team is needed.

Our study had several limitations. The compensation data were not from a random survey, so selection bias was possible if physicians with higher or lower incomes were more likely to respond. Not all specialties admit graduating seniors to training programs in year 1, limiting the number of specialties we can include.

In conclusion, we again found a strong positive correlation between the salary for a specialty and the percentage of year 1 positions filled by graduating US allopathic seniors. We add to previous work by finding a negative correlation between US osteopathic seniors and international medical graduates, suggesting a bias against those groups that is not appropriate based on objective data on the performance of osteopathic and international physicians in the workforce.

## Data Availability

Data are available upon reasonable request from the authors
